# Effects of Kangfuxin on periodontal and masticatory outcome in periodontitis patients during implant supported rehabilitation

**DOI:** 10.3389/fmed.2026.1798184

**Published:** 2026-04-23

**Authors:** Jiaqing Sun, Hao Zhou

**Affiliations:** 1 Dentistry, Tongxiang First People's Hospital, Tongxiang, Zhejiang, China; 2 Department of Pharmacy, Tongxiang First People's Hospital, Tongxiang, Zhejiang, China

**Keywords:** chronic periodontitis, dental implants, Kangfuxin solution, masticatory function, peri-implant health

## Abstract

**Background:**

Chronic periodontitis increases peri-implant disease risk and compromises masticatory function. Kangfuxin solution, a traditional Chinese medicine with anti-inflammatory properties, has shown efficacy in periodontal therapy but remains unevaluated in peri-implant disease prevention. This study assessed its 6-month effects on peri-implant health and masticatory function.

**Methods:**

This retrospective cohort included 300 chronic periodontitis patients (mean age 52.3 ± 8.7 years; 56% male) with partial edentulism who received implant rehabilitation with adjunctive Kangfuxin solution for a minimum of 6 weeks between January 2019 and June 2024. Primary outcomes were changes in probing depth (PD) and clinical attachment level (CAL). Secondary outcomes included peri-implantitis incidence, marginal bone loss (MBL), bite force, mixing ability index (MAI), and inflammatory biomarkers (*n* = 80).

**Results:**

At 6 months, mean PD decreased from 4.8 ± 0.6 mm to 3.4 ± 0.5 mm (*p* < 0.001) and CAL from 5.1 ± 0.8 mm to 4.0 ± 0.7 mm (*p* < 0.001). Peri-implantitis incidence was 3.0% (vs. 12% historical benchmark, *p* = 0.001). MBL averaged 0.28 ± 0.12 mm (94.7% < 0.5 mm). Bite force improved by 39.6% (*p* < 0.001) and MAI from 48.3 ± 9.2 to 61.4 ± 7.8 (*p* < 0.001). IL-1β, IL-6, and TNF-α decreased significantly (all *p* < 0.001). Severe periodontitis and prolonged Kangfuxin exposure (≥9 weeks) showed superior PD reduction. Adverse events were minimal (1.3% mild mucosal irritation); implant survival was 99.6%.

**Conclusions:**

Adjunctive Kangfuxin solution significantly improved peri-implant parameters and masticatory function at 6 months with favorable safety. Randomized trials are warranted to establish its role in high-risk implant patients.

## 1 Introduction

Chronic periodontitis affects approximately 46% of adults in United States and represents the leading cause of tooth loss in individuals over 35 years (1, 2). Characterized by progressive destruction of periodontal attachment and alveolar bone (3), this disease compromises both dentition integrity and masticatory function. Despite advances in regenerative therapies, many patients progress to terminal dentition or partial edentulism requiring prosthetic rehabilitation (4).

Dental implant therapy has emerged as the preferred treatment for replacing missing teeth in periodontally compromised patients, providing significantly better masticatory efficiency and long-term stability than conventional removable prostheses (5, 6). Nevertheless, the long-term success of implants in chronic periodontitis patients is challenged by dysregulated host inflammatory response, altered microbiome composition, and compromised wound healing capacity, collectively increasing susceptibility to peri-implant diseases (7, 8). Systematic reviews report a three-fold higher risk of peri-implantitis in this population, with incidence rates of 15%−28% within 5 years (9). This heightened risk underscores the need for adjunctive therapeutic strategies that modulate the inflammatory microenvironment and enhance peri-implant tissue stability.

Kangfuxin solution, a traditional Chinese medicine preparation derived from Periplaneta americana, demonstrates potent anti-inflammatory, tissue regenerative, and antimicrobial properties (10, 11). Originally developed for wound healing and ulcer treatment, it has gained attention in dentistry for improving outcomes in gingivitis and periodontitis as an adjunct to scaling and root planning (12, 13). Its active components, including peptides and polysaccharides, suppress pro-inflammatory cytokines while promoting fibroblast proliferation and collagen synthesis (14). Mechanistic studies have demonstrated that Kangfuxin promotes angiogenesis-mediated socket healing through upregulation of CCL2 in stem cells (15), and exhibits potent anti-inflammatory effects in experimental periodontitis models via suppression of the PI3K-AKT-mTOR signaling pathway (16). Clinical investigations further confirm that Kangfuxin significantly reduces key inflammatory mediators—including IL-1β, IL-6, IL-17, and TNF-α–in gingival crevicular fluid, correlating with improved periodontal parameters (17). These mechanisms suggest potential utility in optimizing the peri-implant microenvironment following implant surgery. Clinical studies evaluating Kangfuxin solution specifically in the context of implant rehabilitation for chronic periodontitis patients remain scarce, with most existing evidence focusing on general peri-implant disease prevention or periodontal therapy without implants (18).

This retrospective cohort study assessed the 6-month clinical effects of adjunctive Kangfuxin therapy on peri-implant periodontal parameters and masticatory function in patients with chronic periodontitis undergoing implant-supported rehabilitation for partial edentulism. We hypothesized that integration of Kangfuxin solution into a standardized peri-operative protocol (comprising pre-operative conditioning, immediate post-operative care, and maintenance phases, detailed in Section 2.3) would significantly reduce probing depths, improve clinical attachment levels, and enhance masticatory performance compared to baseline, while maintaining a favorable safety profile.

## 2 Methods

### 2.1 Study design and setting

This was a single-center, retrospective cohort study conducted at our hospital. No concurrent control group receiving standard implant care without Kangfuxin was included. The study protocol utilized consecutive patient data spanning a 5-year inclusion window (January 2019 to June 2024), with a standardized follow-up assessment at 6 months ± 2 weeks after definitive prosthetic loading.

The study protocol was reviewed and approved by the Institutional Review Board (IRB) of our hospital (Approval No: 2026-055). Given the retrospective nature of the study involving de-identified patient data, the requirement for informed consent was waived. The study adhered to the principles of the Declaration of Helsinki.

### 2.2 Patient selection and eligibility criteria

Patients were identified through electronic medical record (EMR) queries of the hospital's integrated dental implant database. Inclusion criteria: (1) age 35–70 years; (2) partial edentulism requiring implant-supported fixed rehabilitation of ≥2 contiguous missing posterior teeth (Kennedy Class I or II); (3) chronic periodontitis defined as probing depth (PD) ≥4 mm at ≥30 % of sites and CAL ≥3 mm (16); (4) able/willing to use Kangfuxin solution (no cockroach allergy); (5) complete baseline (T0), pre-surgical (T1) and 6-month post-loading (T2, ±2 weeks) records; (6) implants in functional loading ≥6 months by T2. Exclusion criteria: (1) aggressive periodontitis, uncontrolled diabetes (HbA1c >7.5 %), or systemic conditions impairing healing (e.g., immunosuppression, uncontrolled rheumatoid arthritis); (2) pregnancy/lactation; (3) smoking >20 cigarettes/day or quit < 2 years; (4) severe bruxism, untreated temporomandibular disorder or occlusal trauma; (5) extensive bone augmentation (guided bone regeneration ≥3 walls or graft >1 ml); (6) primary stability < 35 Ncm; (7) follow-up attendance < 80 % or >20 % missing primary outcomes.

### 2.3 Intervention protocol: Kangfuxin solution adjunctive therapy

Kangfuxin solution (KFX, Xinjiang Shengfa Pharmaceutical; 50 ml/bottle, Periplaneta americana extract 0.1 % w/v) was integrated into a standardized peri-operative protocol.

Phase 1: pre-operative conditioning. Following full-mouth supra- and sub-gingival debridement, patients rinsed with 10 ml KFX (diluted 1:1 with sterile water) for 5 min, three times daily, during the 2–4 weeks preceding implant placement.

Phase 2: immediate post-operative care. Post-operatively, rinsing was withheld for 14 days to protect the initial mucosal seal, re-initiated at week 2 and continued at 10 ml twice daily for 4 weeks.

Phase 3: maintenance phase. An intermittent maintenance phase was then prescribed: 10 ml twice daily for 14 consecutive days per month, repeated over 3 months (target cumulative post-operative exposure 12 weeks).

Actual exposure time was recorded to the nearest day from electronic prescription logs, nursing entries and bottle-weight verification; participants were subsequently categorized as < 6, 6– < 9 or ≥9 weeks of cumulative KFX use. Systemic antibiotics were limited to prophylactic amoxicillin-clavulanate 875/125 mg once daily for 3 days post-surgery. Chlorhexidine 0.12 % was prohibited throughout the study period to avoid confounding anti-plaque effects.

### 2.4 Surgical and prosthetic protocol

All patients underwent cone beam computed tomography (KaVo 3D eXam) to assess alveolar morphology, followed by digital planning in coDiagnostiX for stereolithographic guide fabrication. Implantation was performed 8–12 weeks after extraction, once mucosal healing and socket integrity were confirmed. Two board-certified periodontists raised full-thickness flaps and inserted titanium implants (Straumann BLX or NobelActive; Ø 3.5–4.8 mm, length 10–14 mm) at 35–45 Ncm insertion torque under a restorative-driven protocol. Standard post-operative analgesia and anti-inflammatory medication were prescribed. After 3–6 months, osseointegration was verified by resonance frequency analysis (ISQ >70) and provisional prostheses delivered. Following a further 2–3 months of soft-tissue maturation, definitive screw-retained zirconia–ceramic fixed prostheses were produced via a fully digital workflow (3 Shape TRIOS; Ceramill CAD/CAM) with mutually protected occlusion.

### 2.5 Outcome assessment and data collection

Data were retrospectively extracted from the hospital's integrated EMR, PACS, and department registry by two independent investigators using a standardized eCRF; discrepancies were resolved by consensus with a senior periodontist.

Primary outcomes: (i) mean change in probing depth (ΔPD) at implant sites from baseline to 6 months; (ii) 6-month peri-implantitis incidence according to 2017 World Workshop criteria (19), operationalized as simultaneous presence of: (i) probing depth ≥ 5 mm; (ii) bleeding on probing and/or suppuration; and (iii) radiographic marginal bone loss ≥ 0.5 mm.

Secondary endpoints: clinical attachment level (CAL); modified sulcus bleeding index (mSBI) and plaque index (PLI, modified Löe & Silness); full-mouth bleeding score (FMBS) and full-mouth plaque score (FMPS, %); marginal bone loss measured on digital periapical radiographs (Rinn holders, long-cone parallel technique) using ImageJ software (v1.53) with reference landmarks from implant platform to first bone-to-implant contact, calibrated against known implant diameter; intra- and inter-examiner reliability assessed by intraclass correlation coefficient (ICC >0.90), with examiners blinded to Kangfuxin exposure duration; maximum bite force (GM10 transducer, calibrated with 5-kg standard weight before each session; mean of three recordings with 1-min intervals); Masticatory performance was assessed by two-color wax tablet test (20 chewing strokes, standardized 5 × 5 mm bicolour wax tablets, image analysis by trained examiner; intra-class correlation coefficient 0.92 for inter-rater reliability). Interleukin (IL)-1β, IL-6 and TNF-α were quantified with Luminex 200 multiplex assay (Luminex Corporation, Austin, TX, USA) in 80 archived peri-implant sulcular fluid (PISF) samples collected at baseline and 6-month follow-up, stored at −80 °C until batch analysis. Gingival crevicular fluid (GCF) from adjacent natural teeth was sampled in a subset (*n* = 20) for comparison. Sample size was determined by assay cost constraints and availability of paired biobank specimens. Patient-reported treatment satisfaction was evaluated using a 10-point Visual Analog Scale (VAS), with scores ranging from 0 (completely dissatisfied) to 10 (completely satisfied), assessed at baseline and 6-month follow-up.

### 2.6 Statistical analysis

Statistical analyses were conducted in Stata/MP 17.0 and R 4.3.1; two-sided *P* < 0.05 indicated significance. Continuous variables are presented as mean ± SD or median (IQR), and categorical variables as *n* (%). Normality was examined with the Shapiro–Wilk test. The primary endpoint, ΔPD, was analyzed with a paired *t*-test (normal distribution) or Wilcoxon signed-rank test (non-normal) and compared with the minimal clinically important difference of 0.5 mm. Longitudinal changes in PD, CAL and MBL were modeled with linear mixed-effects models including fixed effects for time, age, sex, smoking status and baseline disease severity and random patient intercepts. The cumulative incidence of peri-implantitis is reported with point estimates and Wilson 95 % CIs; an exact binomial test was used to evaluate whether the incidence was below the historical 9%−15 % benchmark, using 5 % as the reference. Correlation between MAI and VAS was assessed with Spearman's coefficient. Multivariable logistic regression (entry *P* < 0.10) identified predictors of mSBI ≥ 2 at 6 months. Pre-specified subgroups were periodontal severity, KFX exposure duration and smoking status. Missing data were handled by multiple imputation (MICE, 20 imputations) and inverse probability weighting for sensitivity analyses; inter-rater reliability (Cohen's κ) for independent duplicate abstraction and the full covariate set included in final multivariable models are detailed in Supplementary Table 1. Comparisons of gingival crevicular fluid biomarkers were adjusted with Bonferroni–Holm correction; the significance threshold was set at α = 0.0125.

## 3 Results

### 3.1 Patient flow and baseline characteristics

**Figure 1:**
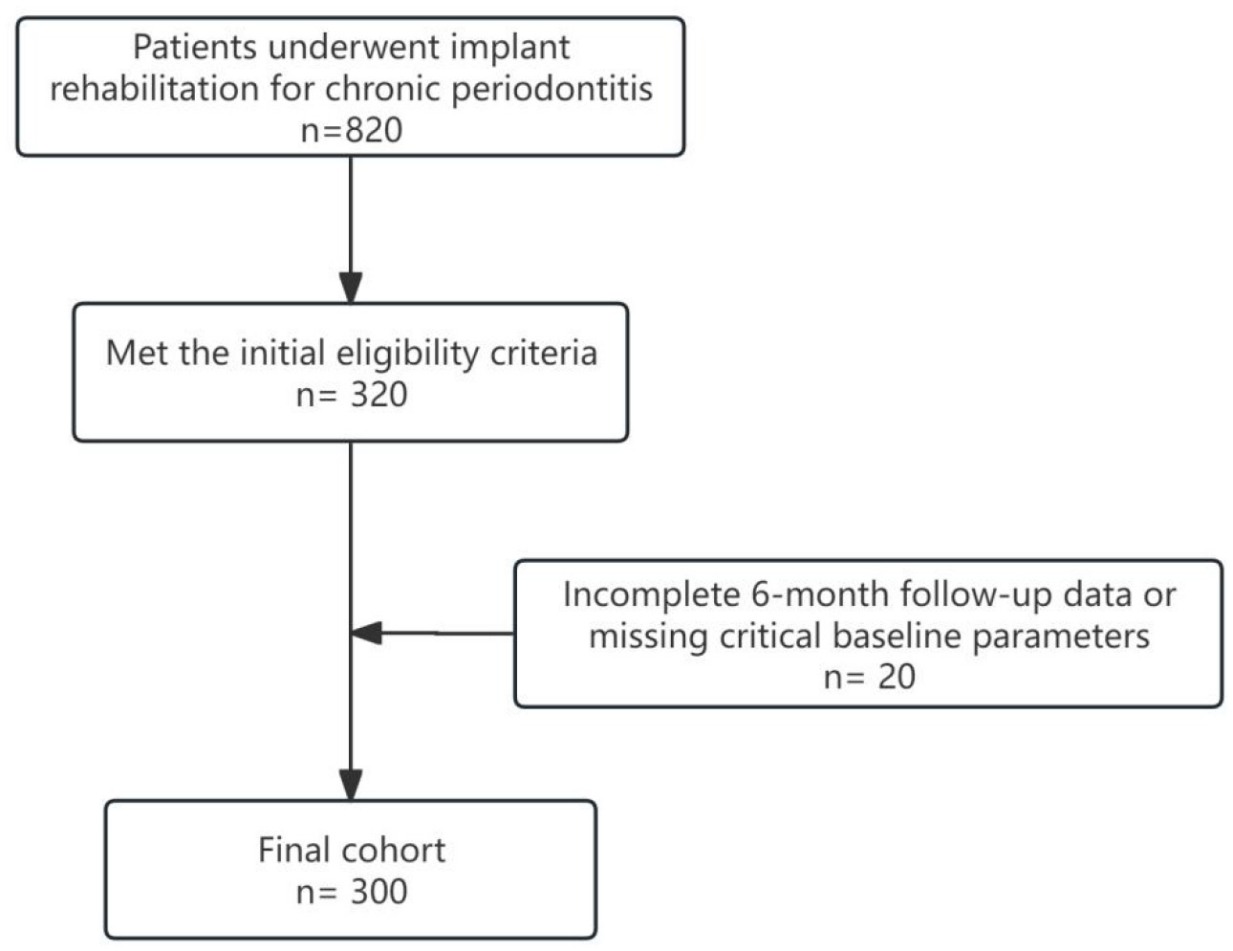
Patient enrollment and screening process.

A total of 820 patients who underwent implant rehabilitation for chronic periodontitis between January 2019 and June 2024 were screened, of whom 320 met the initial eligibility criteria. After excluding 20 patients with incomplete 6-month follow-up data or missing critical baseline parameters, 300 patients were included in the final analytic cohort (Figure 1).

The mean age was 52.3 ± 8.7 years, with 168 males (56.0%) and 132 females (44.0%). Moderate chronic periodontitis was diagnosed in 192 patients (64.0%), while 108 patients (36.0%) presented with severe disease. The mean baseline (T0, prior to periodontal treatment) full-mouth probing depth was 4.8 ± 0.6 mm, clinical attachment level was 5.1 ± 0.8 mm, and the full-mouth bleeding score was 42.3 ± 12.5%. All patients received a cumulative Kangfuxin solution exposure of at least 6 weeks, with a mean exposure duration of 8.4 ± 1.2 weeks. A total of 832 implants were placed, with an average of 2.8 implants per patient, pre-dominantly in the posterior mandible (58.3%). The 6-month follow-up data were complete for 285 patients (95.0%), with 15 patients (5.0%) lost to follow-up or having incomplete clinical records (Table 1).

**Table 1:** Baseline demographic and clinical characteristics of the study cohort.

Characteristic	Mean ±SD or *n* (%)	Range
Age (years)	52.3 ± 8.7	35–70
Sex		
Male	168 (56.0)	—
Female	132 (44.0)	—
Periodontitis severity		
Moderate	192 (64.0)	—
Severe	108 (36.0)	—
Baseline PD (mm)	4.8 ± 0.6	3.5–6.8
Baseline CAL (mm)	5.1 ± 0.8	3.2–7.5
Full-mouth bleeding score (%)	42.3 ± 12.5	18–78
Full-mouth plaque score (%)	38.6 ± 11.2	15–72
Actual KFX exposure 8.4 ± 1.2 weeks (6–12); < 6 w 4 %, 6– < 9 w 24 %, ≥9 w 72 %	8.4 ± 1.2	6–12
Number of implants per patient	2.8 ± 0.6	2–4
Implant location		
Posterior mandible	485 (58.3)	—
Posterior maxilla	347 (41.7)	—
Follow-up completeness at 6 months	285 (95.0)	—

### 3.2 Primary outcome: probing depth reduction

Mean peri-implant probing depth fell from 4.8 ± 0.6 mm at baseline to 3.4 ± 0.5 mm 6 months post-loading (ΔPD = 1.4 ± 0.6 mm; 95 % CI 1.32–1.48; *p* < 0.001), surpassing the minimal clinically important difference of 0.5 mm. Linear mixed-effects modeling showed a significant fixed effect of time (β = −1.42, SE 0.03, *p* < 0.001) and no time × baseline disease-severity interaction. The percentage of patients with PD ≤ 3 mm at all implant sites rose from 0 % at baseline to 67.3 % (202/300) at 6 months; detailed changes in other clinical indices are presented in Tables 2, 3.

**Table 2:** Changes in primary and key periodontal parameters from baseline to 6 months.

Parameter	Baseline (*n* = 300)	6 months (*n* = 285)	Mean Δ (95% CI)	*p*-value
Peri-implant PD (mm)	4.8 ± 0.6	3.4 ± 0.5	1.4 (1.32–1.48)	< 0.001
Peri-implant CAL (mm)	5.1 ± 0.8	4.0 ± 0.7	1.1 (1.02–1.18)	< 0.001
Sites with PD ≤ 3 mm (%)	0 (0)	202 (67.3)	67.3 (61.8–72.8)	< 0.001
mSBI score	2.3 ± 0.5	0.8 ± 0.4	1.5 (1.42–1.58)	< 0.001
PLI score	1.8 ± 0.4	0.9 ± 0.3	0.9 (0.84–0.96)	< 0.001
FMBS (%)	42.3 ± 12.5	18.7 ± 8.9	23.6 (21.8–25.4)	< 0.001
FMPS (%)	38.6 ± 11.2	20.4 ± 7.6	18.2 (16.5–19.9)	< 0.001

**Table 3:** Distribution of probing depth changes (ΔPD) at patient level.

ΔPD category	*n* (%)	Mean ΔPD (mm)
ΔPD ≥2.0 mm	68 (22.7)	2.3 ± 0.3
1.5 ≤ ΔPD < 2.0 mm	145 (48.3)	1.7 ± 0.2
1.0 ≤ ΔPD < 1.5 mm	67 (22.3)	1.2 ± 0.1
0.5 ≤ ΔPD < 1.0 mm	15 (5.0)	0.7 ± 0.1
ΔPD < 0.5 mm	5 (1.7)	0.3 ± 0.1

### 3.3 Secondary clinical and co-primary binary outcomes

Of 300 patients, 9 (3.0 %; 95 % CI 1.4–5.6 %) developed peri-implantitis at 6 months, a rate significantly lower than the 12 % historical benchmark reported for chronic periodontitis patients within 5 years post-implantation (9) (exact binomial *p* = 0.001). Peri-implant CAL fell from 5.1 ± 0.8 mm to 4.0 ± 0.7 mm (Δ = 1.1 ± 0.5 mm; 95 % CI 1.02–1.18); mSBI and PLI declined by 1.5 and 0.9 units, respectively (both *p* < 0.001). Full-mouth bleeding and plaque scores decreased by 23.6 % and 18.2 %, respectively (both *p* < 0.001).

Mean marginal bone loss was 0.28 ± 0.12 mm (95 % CI 0.26–0.30), with 94.7 % of implants showing < 0.5 mm loss; no implant lost > 1.0 mm. Maximum bite force rose from 142.3 ± 28.6 N to 198.7 ± 31.2 N (Δ = 56.4 N; 39.6 % gain; *p* < 0.001), approaching values reported for healthy dentate subjects. MAI improved from 48.3 ± 9.2 to 61.4 ± 7.8 (Δ = 13.1; *p* < 0.001), and VAS satisfaction increased from 4.2 ± 1.3 to 7.8 ± 1.1 (*p* < 0.001). Objective MAI gain correlated moderately with subjective VAS improvement (ρ = 0.54, *p* < 0.001; Table 4). In the biomarker subset (*n* = 80), IL-1β, IL-6 and TNF-α levels fell by 52.6 pg/ml, 26.6 pg/ml and 22.7 pg/ml, respectively (all *p* < 0.001, Bonferroni–Holm adjusted), and IL-1β reduction correlated with PD improvement (*r* = 0.48, *p* < 0.001; Supplementary Table 2).

**Table 4:** Six-month radiographic and functional outcomes.

Outcome	Baseline	6 months	Mean Δ (95 % CI)	*p*-value	Evaluable *n* (%)	Diagnosed *n* (%)^a^
Marginal bone loss (mm)	—	0.28 ± 0.12	—	—	832 (100)	—
Implants with MBL < 0.5 mm	—	788 (94.7)	—	—	832 (100)	—
Implants with MBL 0.5–1.0 mm	—	44 (5.3)	—	—	832 (100)	—
Implants with MBL >1.0 mm	—	0 (0)	—	—	832 (100)	—
Maximum bite force (*N*)	142.3 ± 28.6	198.7 ± 31.2	56.4 (54.1–58.7)	< 0.001	300 (100)	—
MAI score	48.3 ± 9.2	61.4 ± 7.8	13.1 (12.4–13.8)	< 0.001	300 (100)	—
VAS satisfaction (0–10)	4.2 ± 1.3	7.8 ± 1.1	3.6 (3.4–3.8)	< 0.001	300 (100)	—
Patients with peri-implantitis^b^	—	9 (3.0)	3.0 % (1.4–5.6)	0.001	300 (100)	9 (3.0)
Patients with peri-implantitis	0	9 (3.0)	—	0.003	300 (100)	9 (3.0)
Prosthetic complications	—	8 (2.7)	—	—	300 (100)	—

^a^Diagnosed = simultaneous PD ≥5 mm + bleeding/suppuration and marginal bone loss ≥0.5 mm compared with baseline radiograph; only implants with paired readable radiographs were evaluable.

^b^Co-primary endpoint; exact 95% CI 1.4–5.6 %; *p* = 0.001 vs. 12 % historical benchmark for chronic periodontitis patients at 5 years (9).

### 3.4 Subgroup analyses

Subgroup analyses revealed that patients with severe baseline chronic periodontitis (*n* = 108) experienced greater peri-implant PD reduction (1.6 ± 0.5 mm) compared to those with moderate baseline disease (*n* = 192, 1.3 ± 0.6 mm, *p* = 0.002). The duration of KFX exposure also influenced outcomes, with patients using KFX for ≥9 weeks (*n* = 156) showing superior PD improvement (1.5 ± 0.5 mm) vs. those using it for 6–9 weeks (*n* = 144, 1.3 ± 0.6 mm, *p* = 0.01). No significant differences were observed between smokers (*n* = 87) and non-smokers (*n* = 213) in PD reduction (1.4 ± 0.5 mm vs. 1.4 ± 0.6 mm, *p* = 0.89). Age stratification (< 50 years vs. ≥50 years) also showed no significant differential effect (*p* = 0.34; Table 5).

**Table 5:** Subgroup analyses of probing depth reduction at 6 months.

Subgroup	*n*	Baseline peri-implant PD (mm)	ΔPeri-implant PD(mm)	95% CI	*p*-value(interaction)
Periodontitis severity					
Moderate	192	4.6 ± 0.5	1.3 ± 0.6	1.20–1.40	−0.002
Severe	108	5.1 ± 0.6	1.6 ± 0.5	1.48–1.72	
Kangfuxin exposure					
≥9 weeks	216	4.9 ± 0.6	1.5 ± 0.5	1.39–1.61	0.010
6– < 9 weeks	72	4.8 ± 0.6	1.3 ± 0.5	1.15–1.45	
< 6 weeks	12	4.7 ± 0.5	1.1 ± 0.4	0.80–1.40	
Smoking status					
Non-smoker	213	4.8 ± 0.6	1.4 ± 0.6	1.31–1.49	0.89
Smoker	87	4.9 ± 0.6	1.4 ± 0.5	1.26–1.54	
Age					
< 50 years	128	4.8 ± 0.7	1.4 ± 0.6	1.30–1.50	0.34
≥50 years	172	4.9 ± 0.5	1.5 ± 0.5	1.38–1.62	

### 3.5 Safety and adverse events

No serious adverse events related to Kangfuxin solution were reported. Mild oral mucosal irritation was documented in 4 patients (1.3%), which resolved spontaneously within 3 days without discontinuation of the rinse. Two patients (0.7%) experienced transient nausea attributed to the distinctive taste of the cockroach-derived solution, which did not require intervention. Implant failure occurred in 3 implants (0.36%) across 3 patients (1.0%), all attributable to surgical complications (inadequate primary stability) rather than biological factors. All failed implants were replaced, and these patients remained in the final analysis using intention-to-treat principles. Prosthetic complications, primarily minor screw loosening, were observed in 8 prostheses (2.7%) and were resolved with routine maintenance (Table 6).

**Table 6:** Adverse events and complications during 6-month follow-up.

Event	*n* (%)	Severity	Management
Kangfuxin-related adverse events			
Oral mucosal irritation	4 (1.3)	Mild	Self-resolved
Transient nausea	2 (0.7)	Mild	No intervention
Surgical/prosthetic complications			
Early implant failure	3 (1.0)	Moderate	Replacement
Screw loosening	8 (2.7)	Mild	Retightening
Prosthetic chipping	1 (0.3)	Mild	Polishing
Biological complications			
Peri-implant mucositis	23 (7.7)	Mild	Hygiene reinforcement
Peri-implantitis	9 (3.0)	Moderate	Adjunctive therapy
Total adverse events	50 (16.7)	—	—

### 3.6 Sensitivity analyses

Sensitivity analyses confirmed the robustness of primary findings (Table 7). Multiple imputation for 15 patients with missing 6-month data yielded consistent results (ΔPD = 1.39 mm, 95% CI: 1.31–1.47 mm, *p* < 0.001). Inverse probability weighting based on baseline characteristics did not materially alter effect estimates, indicating resilience to selection bias. Per-protocol analysis excluding 3 early implant failures (ΔPD = 1.41 mm, *p* < 0.001) and exclusion of 12 patients with < 6 weeks' KFX exposure (ΔPD = 1.38 mm, 95% CI: 1.30–1.46) both confirmed that results were not driven by protocol deviations or high-adherence subjects alone.

**Table 7:** Sensitivity analyses for primary outcome (ΔPD).

Analysis method	*n*	Mean ΔPD (mm)	95% CI	*p*-value
Primary (complete case)	285	1.40	1.32–1.48	< 0.001
Multiple imputation	300	1.39	1.31–1.47	< 0.001
Inverse probability weighted	300	1.38	1.30–1.46	< 0.001
Per-protocol (excl. failures)	282	1.41	1.33–1.49	< 0.001
Last observation carried forward	300	1.35	1.27–1.43	< 0.001

## 4 Discussion

This retrospective cohort provides preliminary evidence that adjunctive Kangfuxin solution, integrated into a standardized peri-operative protocol, is associated with significant improvements in peri-implant parameters and masticatory function among chronic periodontitis patients undergoing implant rehabilitation. At 6 months, the observed mean PD reduction of 1.4 mm exceeds the measurement error of periodontal probing (typically ±0.5 mm) and aligns with the treatment endpoint target (PPD ≤ 4 mm without BOP) established by the 2017 World Workshop on Periodontal and Peri-Implant Diseases and Conditions (20). This magnitude of improvement is particularly remarkable given that all enrolled patients presented with moderate-to-severe chronic periodontitis, a population typically characterized by refractory inflammatory responses and compromised healing capacity that pre-dispose to implant complications.

The 99.6% implant survival rate and marginal bone loss of 0.28 mm observed in our cohort compare favorably with published benchmarks for periodontally compromised patients, who typically exhibit 5-year survival rates of 92%−95% and annual bone loss exceeding 0.2 mm (21, 22). These findings suggest that Kangfuxin-derived bioactive compounds—including peptides and polysaccharides—may attenuate excessive inflammatory signaling in the peri-implant microenvironment, potentially mitigating the biological burden imposed by the chronic periodontitis phenotype and creating favorable conditions for osseointegration (16, 23).

The significant reductions in clinical attachment level, sulcus bleeding index, and plaque scores corroborate previous investigations of Kangfuxin as an adjunct to conventional periodontal therapy. A recent systematic review reported that Kangfuxin rinses significantly enhanced PD reduction compared to scaling and root planing alone in chronic gingivitis patients (24). Our study extends these benefits to the peri-implant context, where inflammatory cascades are amplified by surgical trauma and foreign body reactions (25, 26). The observed 3.0% peri-implantitis incidence represents a marked reduction compared to the 8.6%−22% reported incidence in chronic periodontitis cohorts receiving standard maintenance (27, 28). This protective effect likely stems from Kangfuxin's ability to suppress IL-1β, IL-6, and TNF-α–pro-inflammatory cytokines that drive osteoclast activation and collagen degradation—potentially via modulation of TLR4/NF-κB signaling pathways (17, 29, 30). The significant correlation between cytokine reduction and PD improvement (*r* = 0.48 for IL-1β) provides mechanistic plausibility, although the retrospective design precludes definitive causal inference.

Functional rehabilitation outcomes were equally noteworthy. Maximum bite force increased by 39.6% from baseline, reaching 198.7 N at 6 months—values comparable to those reported for healthy middle-aged dentate adults (31). Similarly, the mixing ability index improved by 27.1%, restoring masticatory performance to levels comparable with patients possessing intact natural dentitions (32). These gains address the nutritional compromise and quality-of-life deficits previously associated with periodontitis-induced masticatory dysfunction (33), with the parallel improvement in objective (MAI) and subjective (Visual Analog Scale, VAS) measures underscoring the holistic benefit of combining prosthetic rehabilitation with biological modulation.

Subgroup analyses revealed two notable patterns. First, patients with severe baseline periodontitis exhibited greater PD reduction (1.6 mm) than those with moderate disease (1.3 mm, *p* = 0.002), suggesting that higher inflammatory burden provides greater scope for pharmacological modulation. Second, ≥9 weeks of Kangfuxin exposure yielded superior PD improvement compared to 6– < 9 weeks (1.5 mm vs. 1.3 mm, *p* = 0.01). This exposure-duration effect likely reflects two non-mutually exclusive mechanisms: (i) a pharmacodynamic threshold, where sustained bioactive compound exposure during the critical 8–12 week mucosal maturation period achieves therapeutic concentrations for meaningful tissue remodeling (34); and (ii) compliance confounding, as longer exposure correlates with more diligent oral hygiene and follow-up adherence—factors independently associated with peri-implant outcomes (35). Given our fixed rinsing protocol (0.1% w/v, twice daily), we cannot disentangle these effects. Future prospective studies incorporating graded dosing regimens and objective compliance monitoring (e.g., electronic tracking) are warranted to establish minimal effective exposure and characterize potential ceiling effects. Notably, smoking status did not significantly modify treatment response, possibly attributable to the exclusion of heavy smokers (>20 cigarettes/day) from our cohort, thereby limiting generalizability to this high-risk population.

The safety profile of Kangfuxin was favorable, with only 1.3% of patients reporting mild mucosal irritation and no serious adverse events. Despite the cockroach-derived origin (Periplaneta americana), the low discontinuation rate (0.7% due to taste aversion) suggests acceptable real-world tolerability. Combined with low cost and ease of administration, Kangfuxin represents a pragmatic adjunct for resource-limited settings. Nevertheless, the retrospective nature of this study necessitates cautious interpretation, as adverse events may have been under-reported in routine clinical documentation.

Several methodological strengths bolster internal validity. The 5-year inclusion window facilitated assembly of a large, consecutive cohort (*n* = 300) with 95% follow-up completeness, minimizing selection bias. Standardized surgical and prosthetic protocols executed by experienced clinicians ensured procedural homogeneity, while dual independent data extraction (κ>0.85) enhanced data reliability. However, the observed 99.6% survival rate may partly reflect selection bias toward healthier implant candidates within the periodontitis spectrum, given the exclusion of patients requiring extensive bone augmentation (GBR ≥3 walls, indicating complex ridge defects requiring reconstruction of three or more bony walls) or with poor glycemic control (HbA1c >7.5%).

Family physicians serve as critical gatekeepers for patients with chronic periodontitis undergoing implant therapy. Post-referral, general practitioners should: (i) identify high-risk patients (severe periodontitis, uncontrolled diabetes, smokers); (ii) recognize early warning signs (peri-implant swelling, purulence) during routine exams; (iii) optimize systemic health (glycemic control, smoking cessation); and (iv) communicate relevant medications (bisphosphonates, anticoagulants) to dental teams. These strategies reinforce medical-dental integration and support long-term implant success.

Inherent limitations of the retrospective design must be acknowledged. The absence of a parallel control group prevents definitive attribution of benefits to Kangfuxin alone, as improvements could reflect the natural resolution of post-surgical inflammation, the effects of rigorous oral hygiene reinforcement (Hawthorne effect), or regression to the mean, specifically, the rigorous 6-month follow-up protocol with frequent clinical assessments and oral hygiene reinforcement may have independently motivated behavioral improvements, regardless of Kangfuxin exposure. While multivariable adjustment and sensitivity analyses were employed, residual confounding from unmeasured variables—including specific microbiome profiles (e.g., Porphyromonas gingivalis carriage), genetic polymorphisms (e.g., IL-1 gene cluster variants), and precise smoking pack-years—cannot be excluded. Furthermore, the 6-month follow-up, while sufficient for evaluating early healing and short-term peri-implant stability, may be inadequate for definitive assessment of long-term implant survival, crestal bone stability, and peri-implantitis risk. Peri-implant diseases typically manifest over longer periods, with established cohorts reporting progressive incidence increases beyond 12–24 months. Our observed 3.0% incidence should be interpreted as an early indicator rather than a definitive long-term risk estimate. Future prospective studies with ≥3-year follow-up are warranted to validate these preliminary findings and establish Kangfuxin's definitive role in implant therapy. Future studies should incorporate microbiome sequencing and host genotyping to identify personalized response patterns, while cost-utility analyses are warranted to justify integration into public health periodontal programs, particularly in regions with high chronic periodontitis prevalence.

## 5 Conclusion

This retrospective cohort study demonstrates that adjunctive Kangfuxin solution was associated with significant short-term improvements in peri-implant health and masticatory function among chronic periodontitis patients undergoing implant rehabilitation. The robust clinical responses, favorable safety profile, and mechanistic plausibility supported by biomarker data suggest that Kangfuxin may represent a valuable adjunct to conventional peri-implant care. However, these findings require confirmation in randomized trials before evidence-based guidelines can be established for managing high-risk implant patients.

## Data Availability

The original contributions presented in the study are included in the article/Supplementary material, further inquiries can be directed to the corresponding author.
