# Correction: Effects of Kangfuxin on periodontal and masticatory outcome in periodontitis patients during implant supported rehabilitation

**DOI:** 10.3389/fmed.2026.1978727

**Published:** 2026-09-29

**Authors:** Jiaqing Sun, Hao Zhou

**Affiliations:** 1 Dentistry, Tongxiang First People’s Hospital, Tongxiang, Zhejiang, China; 2 Department of Pharmacy, Tongxiang First People’s Hospital, Tongxiang, Zhejiang, China

**Keywords:** chronic periodontitis, dental implants, Kangfuxin solution, masticatory function, peri-implant health

The Ethics statement was erroneously given as “The study protocol was reviewed and approved by the Institutional Review Board (IRB) of our hospital (Approval No: 202500506). Given the retrospective nature of the study involving de-identified patient data, the requirement for informed consent was waived. The study adhered to the principles of the Declaration of Helsinki”. The correct Ethics statement is “The study protocol was reviewed and approved by the Institutional Review Board (IRB) of our hospital (Approval No: 2026-055). Given the retrospective nature of the study involving de-identified patient data, the requirement for informed consent was waived. The study adhered to the principles of the Declaration of Helsinki”.

The original version of this article has been updated.

