# Supplementary material for: Effects of Kangfuxin on periodontal and masticatory outcome in periodontitis patients during implant supported rehabilitation
Source: Front Med (Lausanne). 2026 Apr 23;13:1798184. doi: 10.3389/fmed.2026.1798184 (PMC13149057; doi:10.3389/fmed.2026.1798184)
Supplement: Supplementary file 1 [file Table_1.docx]

**Supplementary Table 1.** Data-abstraction reliability and full covariate set used in multivariable models

| **Variable** | **Categories / Unit** | **Cohen’s κ (95 % CI)** | **Missing n (%)** | **Included in final model** |
| --- | --- | --- | --- | --- |
| Probing depth (mm) | continuous | 0.92 (0.87–0.96) | 0 (0) | Yes |
| Clinical attachment level (mm) | continuous | 0.90 (0.85–0.95) | 0 (0) | Yes |
| Marginal bone loss (mm) | continuous | 0.88 (0.82–0.94) | 0 (0) | Yes |
| Implant surface | SLA / TiUnite / other | 0.91 (0.86–0.95) | 14 (1.7) | Yes |
| Diabetes control | HbA1c <7 % / ≥7 % | 0.86 (0.80–0.92) | 21 (7.0) | Yes |
| Maintenance interval | <4 mo / ≥4 mo | 0.89 (0.84–0.93) | 0 (0) | Yes |
| Smoking status | Never / Former / Current | 0.93 (0.89–0.97) | 0 (0) | Yes |
| Sex | Male / Female | 1.00 | 0 (0) | Yes |
| Age | years | 0.96 (0.93–0.99) | 0 (0) | Yes |
| Baseline periodontal severity | Moderate / Severe | 0.91 (0.86–0.95) | 0 (0) | Yes |

Note: κ calculated from independent duplicate abstraction of 10 % random sample (n = 30 patients, 832 implants).

**Supplementary Table 2.** Changes in Gingival Crevicular Fluid Biomarkers (n=80)

| Biomarker  (pg/ml) | Baseline | 6 months | Mean Δ  (95% CI) | p-value | Correlation with ΔPD |
| --- | --- | --- | --- | --- | --- |
| IL-1β | 142.3 ± 28.4 | 89.7 ± 19.6 | 52.6 (45.2–59.9) | <0.001* | r = 0.48† |
| IL-6 | 67.8 ± 15.2 | 41.2 ± 10.8 | 26.6 (22.8–30.5) | <0.001* | r = 0.41† |
| TNF-α | 58.4 ± 12.6 | 35.7 ± 9.4 | 22.7 (19.4–26.0) | <0.001* | r = 0.38† |

†Spearman correlation, all p<0.001;*Bonferroni-Holm adjusted p < 0.0125.
